# The association between the thyroid feedback quantile-based index and serum uric acid in U.S. adults

**DOI:** 10.1186/s40001-023-01214-3

**Published:** 2023-07-27

**Authors:** Haitao Xie, Ning Li, Guowei Zhou, Zhiyuan He, Xiaoqing Xu, Qian Liu, Haiyan Wang, Jie Han, Le Shen, Peng Yu, Jiandong Chen, Xiaohu Chen

**Affiliations:** 1 Department of Cardiology, Affiliated Hospital of Nanjing University of Chinese Medicine, Nanjing, China; 2 Department of Cardiology, Jiangsu Province Hospital of Chinese Medicine, No. 155, Hanzhong Road, Nanjing, 210004 China; 3 First Clinical Medical College, Nanjing University of Chinese Medicine, Nanjing, China

**Keywords:** Thyroid function, Impaired thyroid hormone sensitivity, Thyroid feedback quantile-based index, Serum uric acid

## Abstract

**Objectives:**

Previous studies have shown that there may be a positive correlation between serum uric acid levels and hyperthyroidism. However, the relationship between thyroid function and serum uric acid in healthy people is unclear. This study analyzed the relationship between impaired thyroid hormone sensitivity and serum uric acid levels, and presented them in quantitative form.

**Research design and methods:**

This is a cross-sectional study of 4460 adults (male: 2300; female: 2160) who participated in the National Health and Nutrition Examination Survey (NHANES) from 2007 to 2010. Parameters representing central sensitivity to thyroid hormones were calculated as: thyroid feedback quantile-based index (TFQI_FT4_), thyroid stimulating hormone index (TSHI), and total thyroxine (T4) resistance index (TT4RI); Peripheral sensitivity to thyroid hormone was evaluated by FT3/FT4 ratio. In addition, we have innovated total triiodothyronine (T3) resistance index (TT3RI) and TFQI_FT3_ indexes based on FT3 and TSH. Multiple linear regression models were used to evaluate the correlation between thyroid resistance index and serum uric acid, and the results were presented graphically as smooth curve fittings.

**Results:**

Higher levels of serum uric acid were associated with decreased sensitivity to thyroid hormones in euthyroid individuals. In conjunction with an increase in the thyroid hormone sensitivity index value, uric acid levels gradually increased as well. Furthermore, we found a segmented relationship between TT3RI and serum uric acid changes. The saturation and threshold analyses indicated that 18.85 was the turning point (logarithmic likelihood ratio test = 0.036). When TT3RI < 18.85, the relationship between serum uric acid and TT3RI was not significant [*β*(95% CI) 0.47 (− 0.05, 1.00), *P* = 0.077], but when TT3RI > 18.85, there was a significant rise in serum uric acid with an increase in TT3RI [*β*(95% CI) 3.94 (0.94, 6.95), *P* = 0.010]. A further finding of the interaction test was that impaired thyroid hormone sensitivity and uric acid changes vary among different age groups and BMI levels.

**Conclusions:**

Decreased sensitivity to thyroid hormones was associated with high levels of serum uric acid in people with normal thyroid function. The interaction test shows that different age groups and BMI groups impact the association between impaired thyroid hormone sensitivity and serum uric acid. Furthermore, smooth curve fitting revealed a segmental relationship between TT3RI and serum uric acid levels.

**Supplementary Information:**

The online version contains supplementary material available at 10.1186/s40001-023-01214-3.

## Introduction

Thyroid hormone plays an irreplaceable role in the energy metabolism of the body. The disorder thyroid dysfunction is closely related to the occurrence of a series of metabolic diseases [1, 2]. It has been suggested that patients with subclinical hypothyroidism may suffer from enhanced hyperuricemia due to impaired thyroid hormone sensitivity [3]. However, there has been no research concerning the effects of decreased thyroid hormone sensitivity on serum uric acid in individuals with normal thyroid function. In light of this, it would be necessary to clarify whether or not there is a correlation between them.

Physiologically, thyroid hormones influence serum uric acid levels by affecting purine nucleotide conversion and uric acid excretion [4, 5]. Since the disorder of thyroid hormone and thyroid-stimulating hormone levels is related to the change in serum uric acid concentration, the contradictory phenomenon of these research conclusions is worth further consideration. We can assume that: different individuals may have different degrees of central resistance to thyroid hormones, and this central resistance is manifested in a general decrease in sensitivity to thyroid hormones, which affects the body's uric acid metabolism process, that is, some people are abnormally sensitive to thyroid hormones, while others are less sensitive to thyroid hormones [6]. Thyroid hormone can maintain its stable state through a negative feedback mechanism, central regulation through the hypothalamic–pituitary–thyroid axis (HPT), and peripheral regulation through influencing the metabolic process [7]. In the population with normal thyroid function, compared with the previous single index, such as the observation of thyroid hormone concentration and thyroid stimulating hormone index (TSHI), the combined calculation of TSH and FT4 may provide a better insight to describe the association between thyroid function and serum uric acid level. Laclaustra et al. proposed a new formula to describe the central sensitivity of thyroid hormones [8]: Thyroid Feedback Quantile-based Index (TFQI), which is calculated based on the empirical combined distribution of FT4 and TSH without the occurrence of extreme distribution in thyroid dysfunction. Therefore, this cross-sectional study aimed to investigate the relationship between central thyroid hormone sensitivity and peripheral thyroid hormone sensitivity and serum uric acid concentration in people with normal thyroid function.

## Materials and methods

### Study design and participants

**Fig. 1:**
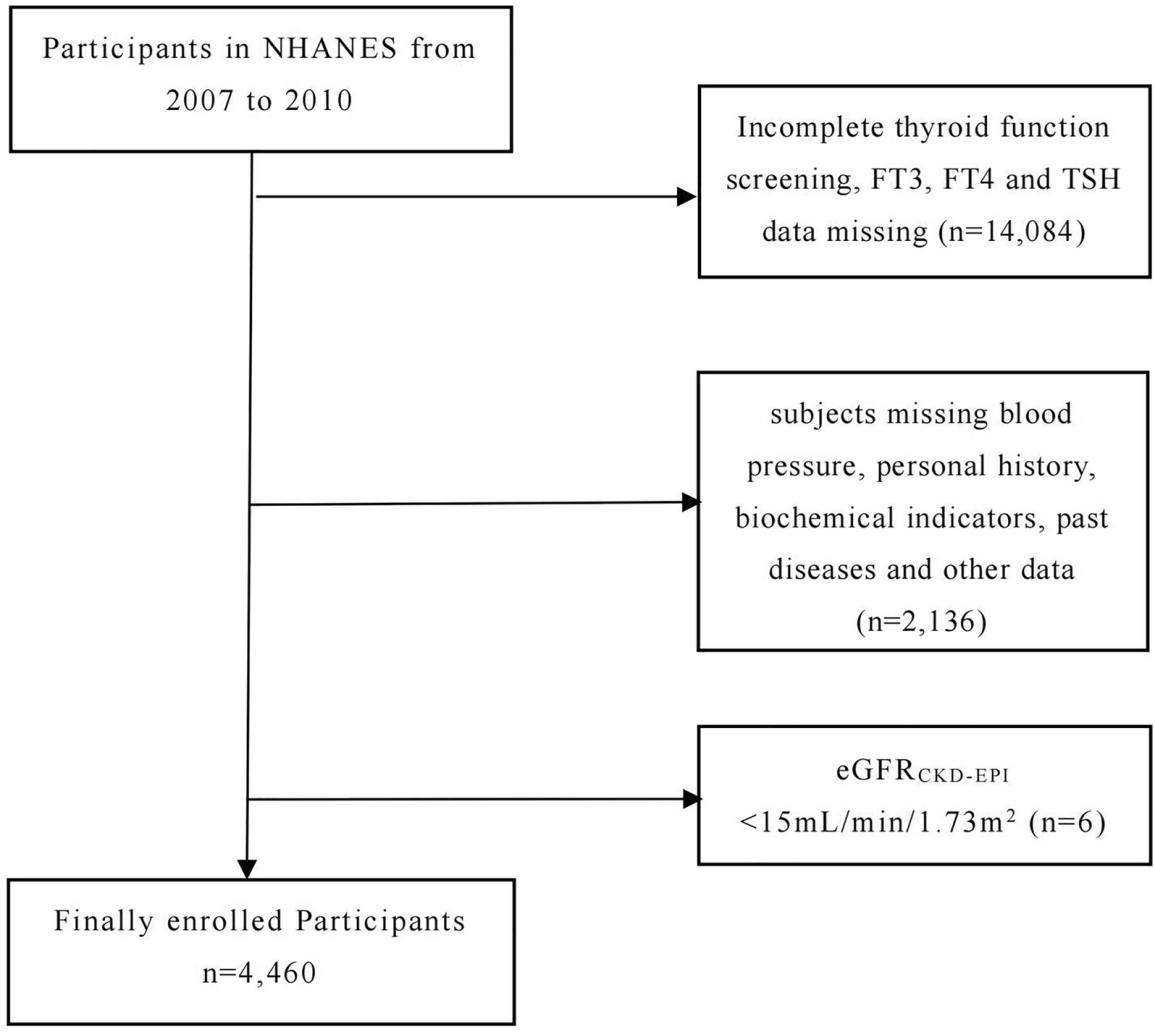
Study design and exclusion information flowchart

For this study, data were derived from the National Health and Nutrition Examination Survey [9]. It is a cross-sectional survey based on the American population that aims to collect information on the health and nutrition status of children and adults across from all walks of life. It is unique in that the interview and physical examination are combined, and a stratified multi-stage sampling design is adopted to obtain representative samples that can reflect the overall population. NHANES began in the early 1960s and is now an ongoing program, sampling a national sample every year of about 5000 people, every 2 years is a survey cycle. All participants obtained approval and written informed consent from the Centers for Disease Control and Prevention (CDC) and the National Center for Health Statistics (NCHS). The study included two NHANES cycles (2007–2008, 2009–2010), involving 20,686 participants. In the end, 4460 people were screened and included in the data analysis. All participants' thyroid function was within the normal range, and those with thyroid dysfunction and thyroid autoimmune diseases were excluded. Below are the specific exclusion criteria: (1) incomplete thyroid function screening, FT3, FT4 and TSH data missing (*n* = 14,084); (2) subjects missing blood pressure, personal history, biochemical indicators, past diseases and other data (*n* = 2136); (3) eGFR_CKD-EPI_ < 15 mL/min/1.73m^2^ (*n* = 6). Details of the study design and exclusions are provided in the flowchart (Fig. 1).

### Laboratory measurements

Blood samples were collected from the venous blood of participants who had fasted for more than 9 h and then were processed and transported to the University of Washington, Seattle, WA for data analysis. Among them, Thyroglobulin antibody (TgAb) and Thyroid peroxidase antibody (TPoAb) were determined by a sequential two-step immunoenzymatic "sandwich" assay. FT4 was determined by a two-step enzyme immunoassay, FT3 was detected by competitive immunoenzyme assay. Thyroid function is normal within the following range: TSH concentrations ranged from 0.34 to 5.60 mIU/L, FT4 concentrations ranged from 7.74 to 20.64 pmol/L, FT3 concentrations ranged from 2.63 to 5.70 pmol/L and thyroid peroxidase antibody concentrations ranged from 0 to 5.61 IU/mL and 0–4.11 IU/mL, respectively. Thyroid dysfunction is defined as TSH and FT4 exceeding the normal laboratory range. The serum uric acid level was measured by timed endpoints, while other biochemical indicators were analyzed by Beckman Synchron LX20 and Beckman UniCel^®^ DxC800 Synchron. These biochemical indicators include urea, creatinine, triglyceride, total cholesterol, high density cholesterol, low density cholesterol, and glycosylated hemoglobin.

### Indexes of thyroid hormone sensitivity

Thyroid Feedback Quantile-based Index (TFQI) can better reflect the response of the pituitary–hypothalamic–thyroid axis (HPT) to changes in peripheral serum FT4 levels, in a continuous manner, deviations from the median pituitary response (inhibition) to thyroid hormone [8]. Other indicators used to evaluate the central sensitivity of thyroid hormones include TSH index (TSHI) [11] and thyrotropin T4 resistance index (TT4RI) [10]. The peripheral sensitivity was reflected by FT3/FT4 ratio [12]. In addition, based on the interaction between TSH and FT3, we innovated two new indicators: TFQI_FT3_, TT3RI, and their respective meanings are similar to TFQI_FT4_ and TT4RI. The value of TFQI_FT4_ ranges from − 1 to 1, with positive values representing a poor sensitivity to thyroid hormones, while negative values reflect a good sensitivity, and 0 indicate a normal sensitivity to thyroid hormone. The TSHI and TT4RI values were negatively correlated with the sensitivity of thyroid center. The thyroid hormone sensitivity indexes are calculated as follows:

1. TSHI = LN [TSH (mIU/L)] + 0.1345 × FT4 (pmol/L) [11];
2. TT4RI = FT4 (pmol/L) × TSH (mIU/L) [10];
3. TFQI_FT4_ = NORMDIST (FT4_cell_in_pmol_per_L, 10.075, 2.155, TRUE) + NORMDIST (LN (TSH_cell_in_mIU_per_L), 0.4654, 0.7744, TRUE) − 1 [8].

### Covariates measurements

#### Blood pressure and body mass index

A mercury sphygmomanometer calibrated with Bowman meters was used to measure the blood pressure of each of the subjects. Following 5 min of sitting, the trained examiner asked the subjects to take three consecutive blood pressure measurements on their right arm, with a 30 s interval between every measurement. Body mass index (BMI) was calculated based on height and weight: BMI = (kg)/(m^2^).

#### Smoking, drinking and physical activity

Each participant was asked to complete a detailed questionnaire survey, which tracked their smoking, drinking, and physical activity, and then was assessed according to the following specific criteria by the interviewer [13]:

1. Do not smoking: who had smoked fewer than 100 cigarettes throughout their lives;
2. Still smoking: currently smoking daily or frequently;
3. Quit smoking: previously smoked, but now do not.
4. Do not drinking: had at most 12 alcohol drinks/lifetime;
5. Drinking occasionally or a lot: had at least 12 alcohol drinks/lifetime or 12 alcohol drinks/1 year.

In this study, all participants completed physical activity questionnaires (PAQ), and the data were analyzed according to the amount of sedentary time, ranging from 0 to 1200 min.

#### Personal medical history

Personal medical history was proposed by the interviewer in the form of a questionnaire using a computer-assisted personal interview (CAPI) system. It was asked of all subjects whether they had been diagnosed by a physician or other health professional with congestive heart failure, coronary heart disease, stroke, diabetes, etc., and for how long they had been ill. In light of the possibility that renal insufficiency may affect uric acid metabolism, we conducted a renal function assessment using the chronic kidney disease epidemiological collaboration (CKD–EPI) equation and excluded participants with an estimated eGFR less than 15 mL/min/1.73 m^2^.

### Statistical analysis

Due to the fact that NHANES does not sample randomly in actual situations, such as oversampling in certain subgroups to increase subgroup sample numbers, leading to unequal sampling probabilities across populations. Therefore, we weighed the samples in accordance with the sample weight guidelines set by the Centers for Disease Control and Prevention of the United States. Survey cycle in this study were 2007–2008 and 2010–2009, as a precaution against overestimated population weights in different cycles, we combine the weights as follows: MEC4YR = 1/2 × WTMEC2YR.

The normal distribution is expressed as weighted mean ± SD for continuous variables, and Analysis of Variance is used to compare groups; if the distribution is skewed, the weighted median (M) is used to describe, and the interquartile range (IQR) is used to describe the degree of dispersion, and the Kruskal–Wallis rank-sum test was used to compare groups. Categorical data were described by rate and composition ratio, and according to the *P* value, comparison between groups was performed using Chi-square test or Fisher's exact test. To test the relationship between thyroid hormone sensitivity indexes and serum uric acid, a multiple linear regression model was developed based on the results of the univariate analysis, and the covariates that had a significant influence (*P* < 0.05) in the univariate analysis were accounted for in the adjustment model as well.

For verification, three models were developed: model 1, non-adjusted covariates, model 2, adjusted for gender, age, and race factors; model 3, based on model 2, added blood pressure, smoking, drinking, BMI, physical activity, urea, creatinine, triglyceride, total cholesterol, high density cholesterol, low density cholesterol, glycosylated hemoglobin, past disease history and eGFR variables. By fitting smooth curves to determine whether there is a piecewise linear relationship between thyroid hormone sensitivity indexes and serum uric acid, we can observe whether there is a threshold effect. To analyze the threshold effect, a piecewise regression model is then employed. Finally, for further evaluation of thyroid hormone sensitivity indexes 's influence on serum uric acid levels, stratified analyses and interaction tests were performed based on gender, age, and BMI. The data were used the statistical software package R (The R Foundation; http://www.r-project.org; version 3.5.3) and EmpowerStats (www.EmpowerStats.com; X&Y Solutions Inc.). *P* value of < 0.05 (double) was considered as statistically significant.

## Results

### Baseline characteristics of participants

This study included 4460 adults with normal thyroid function, mainly non-Hispanic whites, including 2300 males (51.6%) and 2160 females (48.4%), with ages ranging from 20 to 80. Based on TFQI_FT4_, we divided all individuals into four groups. Table 1 shows that, compared with the lowest quartile of TFQI_FT4_, the proportion of males in the highest quartile was higher, as well as blood pressure and BMI levels, furthermore, urea, creatinine, uric acid, low density lipoprotein cholesterol, glycosylated hemoglobin and sedentary time gradually increased, renal function poorer, and the prevalence rates of basic diseases such as congestive heart failure, coronary heart disease, stroke and diabetes were higher. In contrast, the proportion of smoking and drinking gradually decreased (specific data, see Table 1 and Additional file 1: Table S1) (Table 2).

**Table 1:** Baseline characteristics of participants’ general data

TFQIFT4 quartiles	Q1	Q2	Q3	Q4	*P* value
Gender (%)					
Male	50.91	48.94	51.69	52.15	0.673
Female	49.09	51.06	48.31	47.85	
Age	42.84 (41.98,43.70)	44.48 (43.19,45.77)	47.38 (46.00,48.76)	50.36 (49.17,51.56)	< 0.001
Race/ethnicity (%)					
Mexican American	8.8	7.81	7.34	8.29	< 0.001
Other Hispanic	5.92	5.09	4.72	4.27	
Non-Hispanic White	62.86	71.2	73.85	74.25	
Non-Hispanic Black	17.18	11.59	8.07	6.38	
Other race	5.24	4.31	6.02	6.8	
Blood pressure					
Systolic blood pressure	120 (119,122)	121 (119,122)	123 (121,124)	125 (123,126)	< 0.001
Diastolic blood pressure	70 (70,72)	71 (70,72)	72 (71,72)	72 (71,73)	0.232
Smoking status (%)					
Never	49.46	52.34	52.9	54.68	0.022
Current	26.5	23.59	21.22	18.26	
Former	24.05	24.07	25.88	27.06	
Alcohol (%)					0.062
Never	21.57	22.35	23.75	26.14	
Occasionally/a lot	78.43	77.65	76.25	73.86	
Body mass index (kg/m^2^)	28.32 (27.93,28.70)	28.07 (27.46,28.68)	29.33 (28.88,29.78)	29.15 (28.67,29.63)	< 0.001
Physical activity (min)	308 (292,325)	340 (322,358)	350 (332,368)	354 (336,371)	< 0.001
Heart failure (%)					0.143
No	98.63	98.48	98.1	97.34	
Yes	1.37	1.52	1.9	2.66	
Coronary heart disease (%)					< 0.001
No	97.46	98.72	96.66	94.89	
Yes	2.54	1.28	3.34	5.11	
Stroke (%)					0.034
No	97.05	98.05	98.11	96.29	
Yes	2.95	1.95	1.89	3.71	
Diabetes (%)					< 0.001
No	94.67	93.19	91.47	89.04	
Yes	5.33	6.81	8.53	10.96

**Table 2:** Results of single factor analysis

	*β* (95% CI)	*P* value
*Univariate analysis for uric acid*		
Age	0.74 (0.59, 0.87)	< 0.001
Gender	Male ( −)	–
female	− 70.28 (− 74.75, − 65.81)	< 0.001
Race/ethnicity	6.69 (4.37, 9.01)	< 0.001
Systolic blood pressure	0.67 (0.54, 0.79)	< 0.001
Diastolic blood pressure	1.03 (0.81, 1.24)	< 0.001
Smoking	13.15 (10.24, 16.05)	< 0.001
Alcohol	20.38 (14.91, 25.86)	< 0.001
Body mass index (kg/m^2^)	3.49 (3.12, 3.85)	< 0.001
Physical activity (min)	0.03 (0.014, 0.04)	< 0.001
Urea	13.16 (11.78, 14.56)	< 0.001
Creatinine	2.29 (2.17, 2.41)	< 0.001
Total cholesterol	5.79 (3.50, 8.07)	< 0.001
Triglyceride	9.99 (8.37, 11.61)	< 0.001
High density cholesterol	− 46.52 (− 52.48, − 40.55)	< 0.001
Low density cholesterol	10.19 (7.694 12.70)	< 0.001
Glycosylated hemoglobin	2.31 (0.03, 4.59)	0.047
eGFR (mL/min/1.73m^2^)	− 1.26 (− 1.37, − 1.15)	< 0.001
Heart failure	− 55.13 (− 70.31, − 39.94)	< 0.001
Coronary heart disease	− 31.09 (− 43.92, − 18.28)	< 0.001
Stroke	− 27.14 (− 40.47, − 13.81)	< 0.001
Diabetes	11.77 (4.14, 19.39)	0.002
FT3/FT4	43.84 (14.90, 72.78)	0.003
TFQI_FT4_	17.37 (9.69, 25.04)	< 0.001
TFQI_FT3_	27.61 (17.11, 38.10)	< 0.001
TSHI	10.86 (6.57, 15.15)	< 0.001
TT3RI	1.60 (1.07, 2.13)	< 0.001
TT4RI	0.59 (0.35, 0.84)	< 0.001

### Association of thyroid hormone sensitivity indexes with serum uric acid

Multiple linear regression was used to validate the correlation between thyroid hormone sensitivity indexes and serum uric acid. As a result of fully adjusting for potential confounding factors, model 3 showed that thyroid hormone sensitivity index was positively correlated with serum uric acid levels. As an example, TFQIFT4 (i.e., central thyroid hormone sensitivity) increases by 1 unit, while serum uric acid increases by 8.33 μmol/L [TFQI_FT4_: *β*(95% CI) 8.33 (1.79, 14.87), *P* = 0.013]. Similarly, When FT3/FT4 (i.e., peripheral thyroid hormone sensitivity) increases by 1 unit, the serum uric acid level increases by 35.05 μmol/L [FT3/FT4: *β*(95% CI) 35.05 (9.27, 60.83), *P* = 0.007]. It is evident from the above results that serum uric acid levels are associated with thyroid hormone sensitivity impairment. The effects of other thyroid hormone sensitivity indexes are presented in Table 3.

**Table 3:** Association of sensitivity of thyroid hormone indexes with serum uric acid

Model 1			Model 2			Model 3		
Non-adjusted	*β* (95% CI)	*P* value	Adjust I	*β* (95% CI)	*P* value	Adjust II	*β* (95% CI)	*P* value
FT3/FT4	43.84 (14.90, 72.78)	0.003	FT3/FT4	33.44 (6.13, 60.74)	0.014	FT3/FT4	35.05 (9.27, 60.83)	0.007
TFQI_FT4_	17.37 (9.69, 25.04)	< 0.001	TFQI_FT4_	12.75 (5.65, 19.86)	< 0.001	TFQI_FT4_	8.33 (1.79, 14.87)	0.013
TFQI_FT3_	27.61 (17.11, 38.10)	< 0.001	TFQI_FT3_	21.71 (12.07, 31.36)	< 0.001	TFQI_FT3_	9.91 (0.95, 18.86)	0.03
TSHI	10.86 (6.57, 15.15)	< 0.001	TSHI	8.58 (4.59, 12.56)	< 0.001	TSHI	4.02 (0.37, 7.66)	0.031
TT4RI	0.59 (0.35, 0.84)	< 0.001	TT4RI	0.48 (0.25, 0.70)	< 0.001	TT4RI	0.25 (0.04, 0.46)	0.014
TT3RI	1.60 (1.07, 2.13)	< 0.001	TT3RI	1.25 (0.77, 1.73)	< 0.001	TT3RI	0.76 (0.31, 1.21)	< 0.001

Model 2: Adjusted by age, gender, race

Model 3: Blood pressure, smoking, alcohol, BMI, physical activity, urea, creatinine, triglyceride, total cholesterol, high density cholesterol, low density cholesterol, glycosylated hemoglobin, past disease history and eGFR were further adjusted based on model 2

### Smooth curve fitting and threshold effect analysis of thyroid hormone sensitivity indexes and serum uric acid

**Fig. 2:**
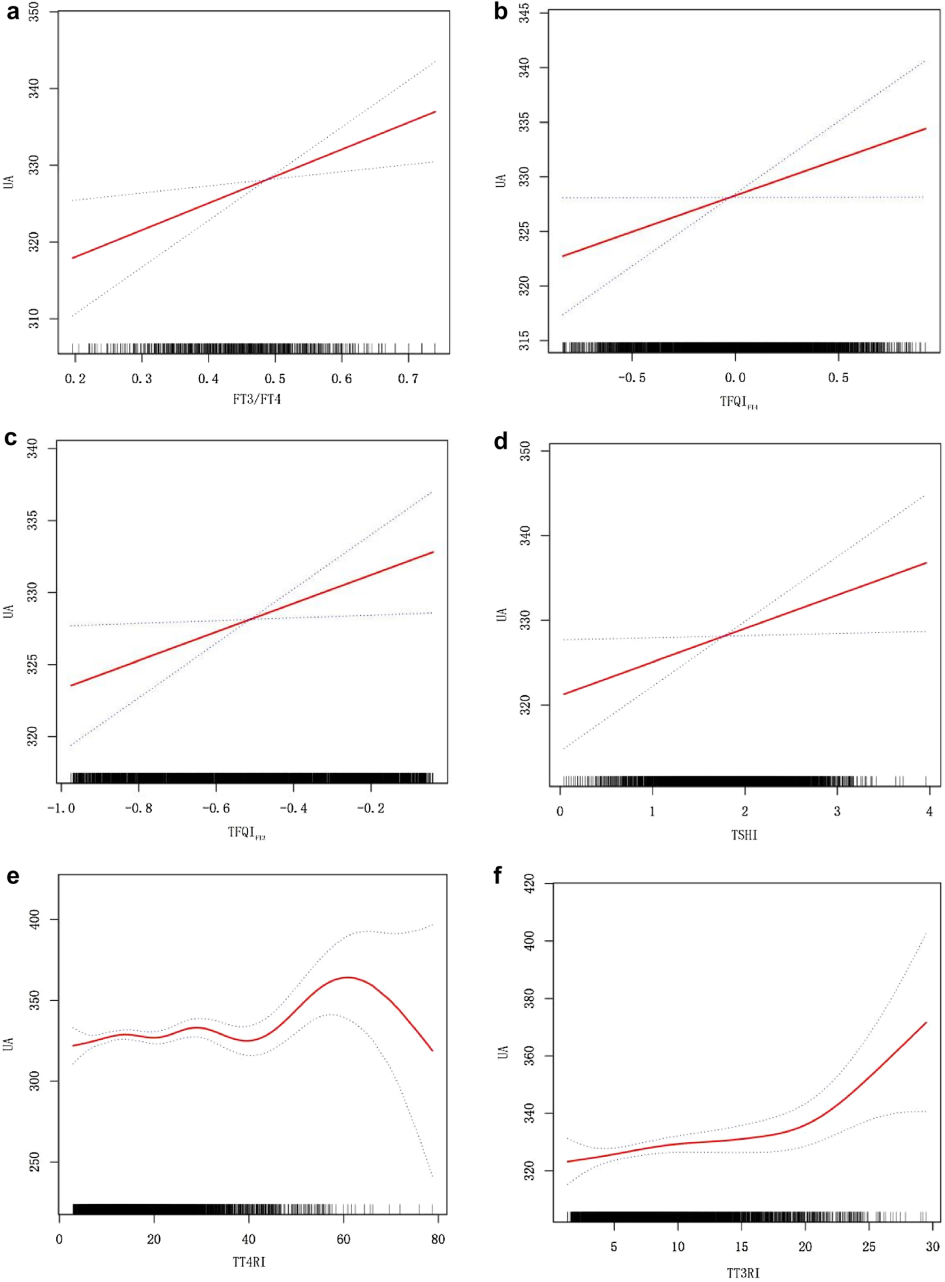
Relationship of thyroid hormone sensitivity index with serum uric acid

Based on the smooth curve fitting graph in combination with the results from model 3, it appears that the level of serum uric acid gradually increases with an increasing thyroid hormone sensitivity indexes value, but between TT4RI and serum uric acid, there is no significant linear relationship may be because *β* = 0.25 is too small (Fig. 2). In the smooth curve fitting analysis, TT3RI and serum uric acid have a segmented relationship after adjusting for confounding factors. Further analysis found that 18.85 was the turning point (logarithmic likelihood ratio test = 0.036). In the absence of TT3RI higher than 18.85, the relationship between serum uric acid and TT3RI was not significant [*β*(95% CI) 0.47 (− 0.05, 1.00), *P* = 0.077], but in the presence of TT3RI higher than 18.85, with an increase of TT3RI, there was a significant rise in serum uric acid [*β*(95% CI) 3.94 (0.94, 6.95), *P* = 0.010] (Table 4).

**Table 4:** Piecewise regression analysis between TT3RI and serum uric acid

The turning point of TT3RI level	Multivariate model	*P* value
	Adjusted *β* (95% CI)	
TT3RI < 18.85	0.47 (− 0.05, 1.00)	0.077
TT3RI ≥ 18.85	3.94 (0.94, 6.95)	0.01

Figure 2a–f show relationship of thyroid hormone sensitivity indexes with serum uric acid.

**Fig. 3:**
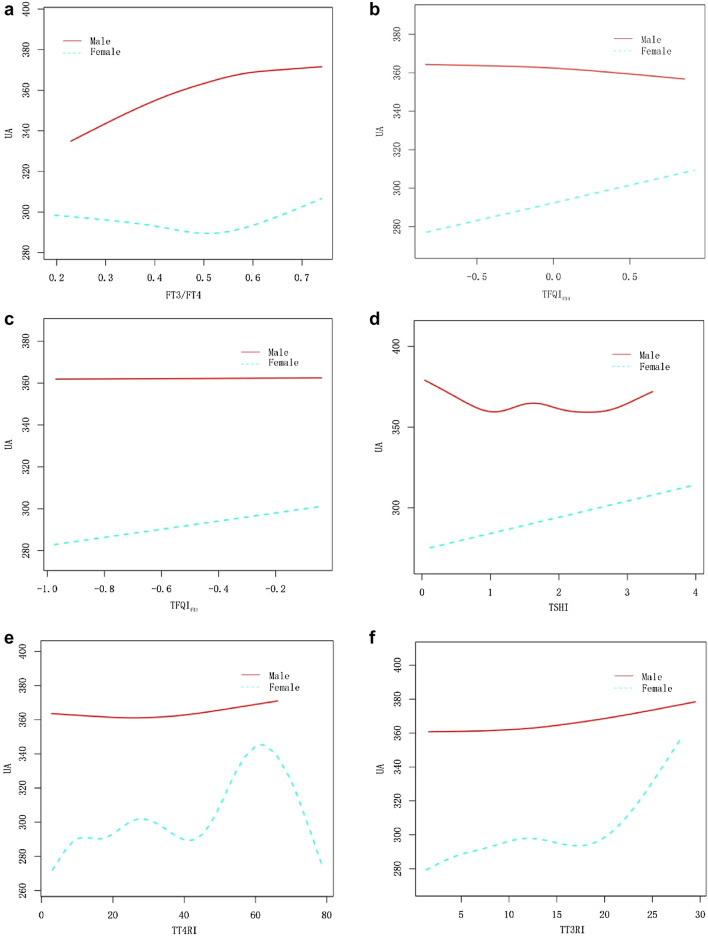
Relationship of thyroid hormone sensitivity indexes with serum uric acid by gender

**Fig. 4:**
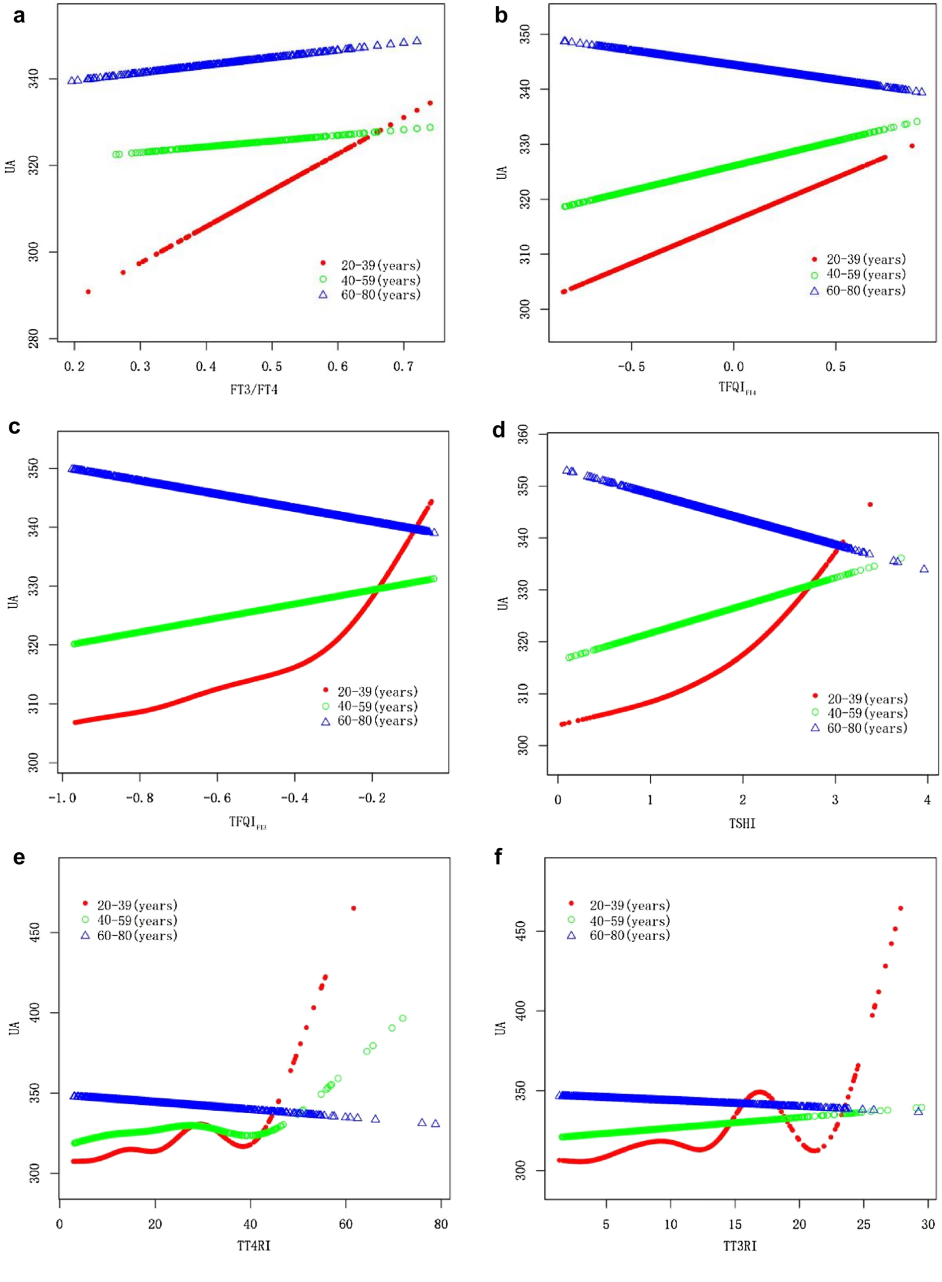
Relationship of thyroid hormone sensitivity indexes with serum uric acid by age

**Fig. 5:**
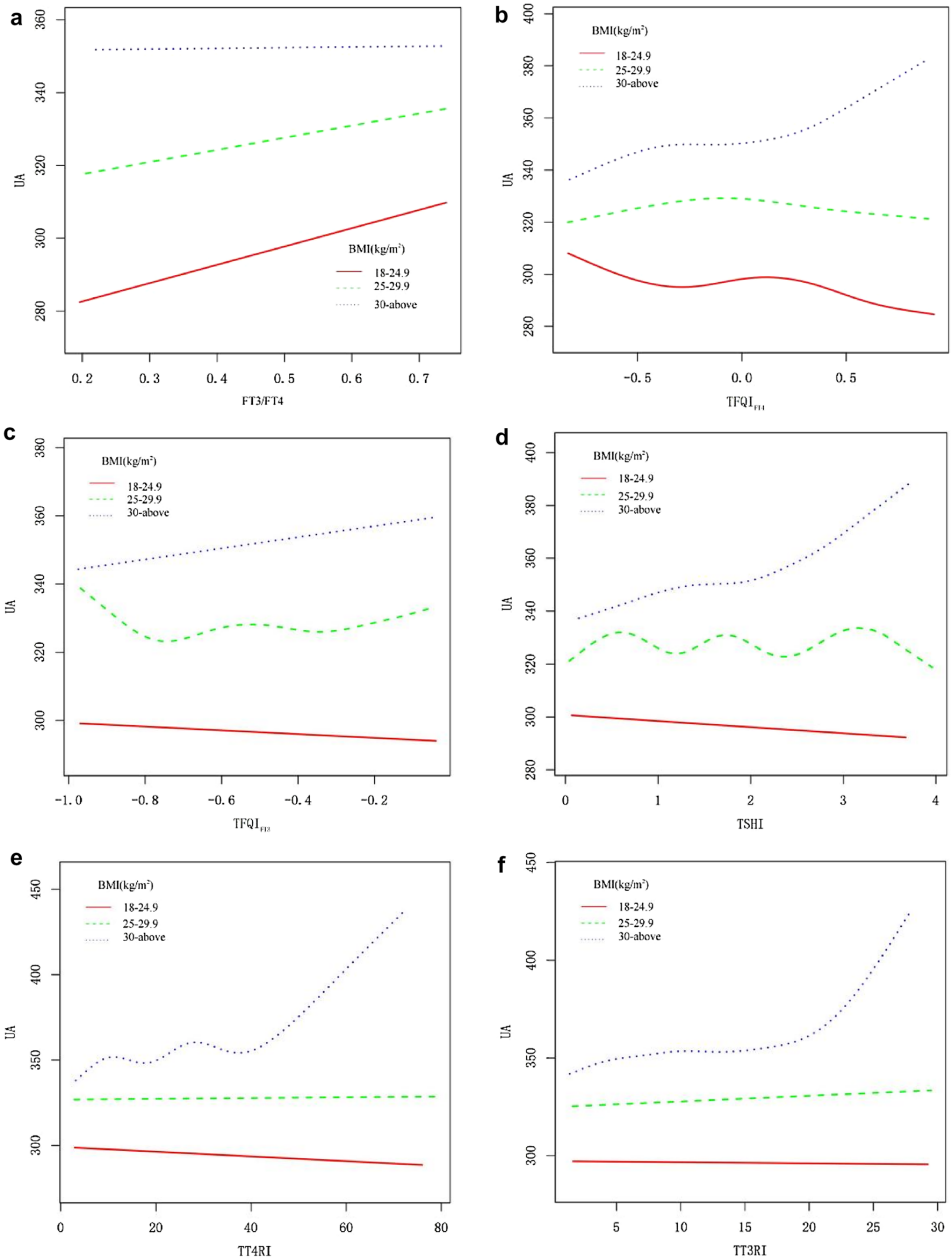
Relationship of thyroid hormone sensitivity indexes with serum uric acid by BMI

Central thyroid hormone sensitivity indexes: thyroid feedback quantile-based index (TFQI_FT4_, TFQI_FT3_), T4 resistance index (TT4RI), T3 resistance index (TT3RI), TSH index (TSHI). The higher values, the lower the central sensitivity to thyroid hormone.

Peripheral thyroid hormone sensitivity index: FT3/FT4.

### Analyses of subgroups and interaction tests

As shown in the subgroup analysis (Table 5), after stratification on gender, age, and BMI, as well as adjustment for other covariables, the interaction test results demonstrated that impaired thyroid hormone sensitivity was associated with a significant difference in serum uric acid among different age groups and BMI classification populations, and that the trend was generally consistent with that for the entire populations (Fig. 3a–f). For example, in obese individuals (BMI ≥ 30), serum uric acid concentration will increase by 15.91 μmol/L for every standard unit of TFQI_FT4_ (*P* for interaction = 0.019) (Fig. 4a–f); in comparison with middle-aged and elderly individuals, young people's (20–39 years) serum uric acid concentration will increase by 17.50 μmol/L for each standard unit of TFQI_FT3_ increase (*P* for interaction = 0.016) (Fig. 5a–f).

**Table 5:** Analyses of subgroups and interaction tests

Subgroups	FT3/FT4	TFQI_FT4_	TFQI_FT3_	TSHI	TT4RI	TT3RI
Gender	Adjusted *β* (95% CI)	–	–	–	–	–
Male	34.75 (− 0.44, 69.93)	− 0.99 (− 10.07, 8.08)	3.43 (− 8.97, 15.83)	− 0.12 (− 5.22, 4.97)	0.08 (− 0.22, 0.37)	0.42 (− 0.19, 1.03)
Female	36.71 (− 0.49, 73.90)	11.97 (2.50, 21.44)*	15.74 (3.09, 28.40)*	7.24 (1.97, 12.51)**	0.36 (0.07, 0.66)*	1.01 (0.37, 1.65)**
*P*-interaction	0.939	0.052	0.171	0.048	0.172	0.187
Age group						
20–39 years	62.94 (18.72, 107.17)**	5.93 (− 5.38, 17.24)	17.50 (2.25, 32.75)*	7.99 (1.88, 14.10)**	0.55 (0.16, 0.94)**	1.38 (0.61, 2.16)***
40–59 years	− 1.46 (− 43.05, 40.12)	9.02 (− 1.91, 19.94)	7.85 (− 6.97, 22.67)	4.53 (− 1.60, 10.66)	0.27 (− 0.09, 0.62)	0.44 (− 0.31, 1.18)
60–80 years	21.42 (− 21.80, 64.63)	− 5.39 (− 16.41, 5.62)	− 12.56 (− 27.31, 2.19)	− 5.30 (− 11.46, 0.87)	− 0.23 (− 0.55, 0.08)	− 0.36 (− 1.09, 0.36)
*P*-interaction	0.107	0.156	0.016	0.025	0.006	0.005
BMI (kg/m^2^)						
18–24.9	30.73 (− 19.56, 81.03)	− 2.28 (− 14.57, 10.01)	− 5.26 (− 21.53, 11.01)	− 1.79 (− 8.51, 4.93)	− 0.12 (− 0.51, 0.26)	− 0.10 (− 0.94, 0.73)
25–29.9	39.78 (− 2.11, 81.67)	− 3.47 (− 14.18, 7.24)	− 1.21 (− 16.07, 13.64)	− 2.17 (− 8.26, 3.92)	− 0.06 (− 0.41, 0.29)	0.17 (− 0.59, 0.93)
≥ 30	0.24 (− 39.40, 39.89)	15.91 (5.41, 26.40)**	15.97 (1.83, 30.10)*	8.72 (2.83, 14.61)**	0.51 (0.18, 0.83)**	1.05 (0.36, 1.73)**
*P*-interaction	0.368	0.019	0.103	0.017	0.018	0.074

*P < 0.05; **P < 0.01; ***P < 0.001

Adjust model adjust for: gender, age, race, blood pressure, smoking, alcohol, BMI, physical activity, urea, creatinine, triglyceride, total cholesterol, high density cholesterol, low density cholesterol, glycosylated hemoglobin, past disease history and eGFR. All the models are not adjusted for the variable itself in each stratification

## Discussion

In this cross-sectional study of 4460 U.S. adults enrolled in the NHANES, we evaluate the association between thyroid hormone sensitivity (both central and peripheral) and serum uric acid levels in people with normal thyroid function. From our results, both central and peripheral thyroid hormone sensitivity indexes, there is a clear relationship between serum uric acid levels. Thyroid hormones and body metabolism have been the subject of controversy in past studies, and so far, the relationship between thyroid hormone and serum uric acid levels in euthyroid individuals has not been well-explained [14, 15]. This creates an obvious disadvantage in the clinical diagnosis and treatment process. Specific data in our study indicate that all indexes of thyroid sensitivity were observed positive correlation with serum uric acid levels. Taking TFQI_FT4_ and FT3/FT4 as examples, where serum uric acid increases by 8.33 μmol/L for every unit increase in TFQI_FT4_ [(95% CI) (1.79, 14.87)), *P* = 0.013]; for each unit increase in FT3/FT4, the level of serum uric acid increases by 35.05 μmol/L [(95% CI) (9.27, 60.83), *P* = 0.007].

This study is the first to assess how impaired thyroid hormone sensitivity affects serum uric acid metabolism from the perspective of thyroid hormone sensitivity in a representative American adult sample, in contrast to previous observations of its effect on serum uric acid concentration only from single indicators, such as FT3 and FT4 [16, 17]. The new thyroid hormone resistance index, TFQI, is based on the empirical joint distribution of FT4 and TSH with the advantage of not yielding extreme values in the case of thyroid gland dysfunction, and can be easily used to calculate any particular individual with reference to the population of the U.S.

Physiologically, thyroid hormones play a significant role in stimulating the metabolism of the human body. As thyroid hormone levels increase, more adenosine triphosphate (ATP) is consumed, leading to higher levels of adenine ribonucleotides, which may further affect purine metabolism and make serum Uric acid levels higher. Studies have shown that serum uric acid is positively correlated with FT3, but not FT4 and TSH levels in individuals with normal thyroid function; nevertheless, other studies have shown that serum uric acid increases with an increase in FT4 levels [14]. Consequently, we conducted this study because of the inconsistency of these findings. In this study, we have conducted a first analysis of the association from the perspective of thyroid hormone sensitivity which may explain some of the inconsistency in previous studies. It is likely that some individuals with normal thyroid function are less sensitive to thyroid hormones, whereas others are normal or more sensitive. Due to the existence of thyroid central resistance, the same level of thyroid hormone may have varying effects on uric acid metabolism in different individuals, thus reconciling the above inconsistent research contradictions.

With an increase in thyroid resistance index, the proportion of obese people increased relative to baseline characteristics. Except for FT3/FT4, all representative thyroid central sensitive indicators were significantly correlated with serum uric acid levels in obese subjects based on the results of subgroup analysis and interaction tests. Previous studies have reported similar findings, namely, the higher the thyroid resistance index, the greater the risk of obesity [18]. A certain stimulation of the thyroid axis may be happening in the state of excess energy metabolism, but the thyroid does not compensate for the increased energy consumption due to its state of excess energy metabolism. As a result of metabolic syndrome, thyroid function can also be affected to some extent. As an example, by acting on TRH neurons via the JAK/STAT signaling pathway, leptin can promote the release of TRH and further increase pituitary TSH secretion [19]. Furthermore, thyroid function may also be affected by low-grade inflammation caused by inflammatory cytokines associated with metabolic syndrome [20]. These factors may collectively result in an expression compatible with resistance to thyroid hormone. There is a high incidence of metabolic syndrome among obese individuals [21, 22]. Hence, changes in serum uric acid concentrations observed in obese individuals may be caused by decreased thyroid hormone sensitivity alone, or by both the metabolic syndrome and thyroid hormone resistance.

Our further consideration of the reasons for the different results based on gender stratified analysis is warranted. During the study, we could not completely eliminate residual confounding effects despite adjusting for age and other potential confounders. For example, a previous study found that TSH and FT4 levels are associated with changes in estradiol concentrations [23]. As a consequence, we should consider whether a compounding effect of sex hormones is responsible for the increase in serum uric acid levels associated with thyroid hormone sensitivity decreased. It is thought that thyroid hormones can regulate the transcription of sex hormone-binding globulin (SHBG), which has different binding affinities with testosterone and estradiol, thereby altering sex hormone levels [24]. However, no link has been found between FT4 and sex hormones in Further Mendelian randomization studies [25]. Typically, premenopausal women have lower serum uric acid levels than men of the same age, and after menopause, the serum uric acid level increases, because sex hormone levels change [26]. Nevertheless, the interaction test showed that the correlation between thyroid hormone sensitivity index and serum uric acid changes was mostly stable between sexes in this study, which indicates reliability of the overall results. Therefore, we speculate that this result is due to a separate effect of heterogeneity within the female subgroup. Until now, no studies have investigated the effect of gender on thyroid hormone and serum uric acid metabolism from the perspective of thyroid hormone sensitivity, and there is still controversy regarding the causal relationship between thyroid function and sex hormones. Thus, further research is needed on thyroid hormone sensitivity and sex hormones in the future.

It was observed that thyroid hormone sensitivity indicators except TFQI_FT4_ were significantly correlated with serum uric acid in the young (20–39 years), but this association did not persist in the middle-aged and elderly. According to cross-sectional studies [27, 28], elderly people have significantly higher TSH levels compared to young people when FT4 is within the normal range and the FT4 concentration remains the same. As we age, the hypothalamus–pituitary–thyroid axis also undergoes complex physiological changes [29]. As a consequence, we speculate that: on one hand, middle-aged and elderly individuals may increase TSH secretion set points to maintain FT4 concentrations due to a decreased sensitivity of negative feedback from thyroid hormones or a decrease in TSH activity. On the other hand, with age, the central thyroid sensitivity may also have some deviations, and deiodinase activity and its balance may also play a role in affecting thyroid hormone sensitivity [30, 31]. Thus, the changes in uric acid levels due to impaired thyroid hormone sensitivity in young individuals will be more pronounced than those in middle-aged and elderly individuals.

## Conclusion

As a result of these findings, we believe that impaired thyroid hormone sensitivity (central or peripheral) is an independent risk factor to elevated serum uric acid levels in the euthyroid individuals, that is, higher values in indices of resistance to thyroid hormone are associated with higher serum uric acid levels. In addition, the interaction test also indicated that the correlation between the two remained relatively stable, and the trend was generally consistent with that for the entire populations. Moreover, we also found a segmental relationship between TT3RI and serum uric acid based on smooth curve fitting, which is a highlight of the paper. In the absence of TT3RI higher than 18.85, the relationship between serum uric acid and TT3RI was not significant, but when TT3RI was higher than 18.85, serum uric acid increased significantly with the increase of TT3RI. It is suggested that the above-mentioned thyroid resistance index can be taken into account when evaluating changes in serum uric acid in the future, which may provide novel insights in clinical diagnosis and treatment.

## Supplementary Information

**Additional file 1. Table S1**: Baseline characteristics of participants' biochemical indicators.

## Data Availability

Publicly available data sets were analyzed in this study. This data can be found here: https://wwwn.cdc.gov/nchs/nhanes/Default.aspx.
