# Supplementary material for: The association between the thyroid feedback quantile-based index and serum uric acid in U.S. adults
Source: Eur J Med Res. 2023 Jul 27;28:259. doi: 10.1186/s40001-023-01214-3 (PMC10373382; doi:10.1186/s40001-023-01214-3)
Supplement: Supplementary file 1 — Additional file 1. Table S1: Baseline characteristics of participants' biochemical indicators. [file 40001_2023_1214_MOESM1_ESM.docx]

Supplementary Table 1. Baseline characteristics of participants' biochemical indicators

| TFQI_FT4_ quartiles | Q1 | Q2 | Q3 | Q4 | *P*-value |
| --- | --- | --- | --- | --- | --- |
| Urea (mmol/L) | 4.26 (4.14 ,4.38) | 4.45 (4.28 ,4.61) | 4.61 (4.47 ,4.74) | 4.88 (4.79 ,4.96) | <0.001 |
| Creatinine (mmol/L) | 73.99 (72.88 ,75.10) | 75.98 (74.47 ,77.49) | 76.41 (74.74 ,78.09) | 78.79 (77.49 ,80.09) | <0.001 |
| Uric acid (umol/L) | 319.02 (313.23 ,324.82) | 325.07 (318.97 ,331.17) | 330.11 (325.03 ,335.18) | 330.23 (324.93 ,335.52) | 0.003 |
| Triglyceride (mmol/L) | 1.80 (1.70 ,1.90) | 1.81 (1.70 ,1.93) | 1.90 (1.74 ,2.05) | 1.82 (1.73 ,1.91) | 0.673 |
| Total cholesterol (mmol/L) | 5.10 (5.03 ,5.17) | 5.14 (5.06 ,5.21) | 5.10 (5.02 ,5.18) | 5.07 (4.98 ,5.15) | 0.743 |
| High density cholesterol (mmol/L) | 1.38 (1.34 ,1.42) | 1.35 (1.31 ,1.39) | 1.33 (1.30 ,1.36) | 1.32 (1.29 ,1.35) | 0.008 |
| Low density cholesterol (mmol/L) | 3.36 (3.30 ,3.42) | 3.42 (3.36 ,3.48) | 3.39 (3.32 ,3.45) | 3.39 (3.31 ,3.46) | 0.635 |
| Glycosylated hemoglobin (%) | 5.50 (5.47 ,5.54) | 5.52 (5.46 ,5.59) | 5.61 (5.56 ,5.67) | 5.68 (5.60 ,5.77) | <0.001 |
| FT4 (pmol/L) | 8.82 (8.71 ,8.93) | 9.59 (9.48 ,9.69) | 10.37 (10.17 ,10.58) | 11.47 (11.35 ,11.59) | <0.001 |
| FT3 (pmol/L) | 4.84 (4.79 ,4.90) | 4.84 (4.80 ,4.87) | 4.85 (4.82 ,4.88) | 4.84 (4.81 ,4.88) | 0.546 |
| TSH (mIU/L) | 1.00 (0.97 ,1.04) | 1.52 (1.47 ,1.57) | 1.94 (1.82 ,2.06) | 2.57 (2.49 ,2.65) | <0.001 |
| TgAb (IU/mL) | 0.68 (0.65 ,0.70) | 0.68 (0.64 ,0.72) | 0.66 (0.63 ,0.68) | 0.66 (0.64 ,0.68) | 0.566 |
| TPOAb (IU/mL) | 0.92 (0.83 ,1.01) | 0.81 (0.75 ,0.88) | 0.83 (0.77 ,0.89) | 0.84 (0.79 ,0.90) | 0.207 |
| FT3/FT4 | 0.55 (0.55 ,0.56) | 0.51 (0.50 ,0.52) | 0.48 (0.47 ,0.49) | 0.43 (0.42 ,0.43) | <0.001 |
| TFQI_FT3_ | -0.72 (-0.73 ,-0.71) | -0.55 (-0.56 ,-0.53) | -0.45 (-0.48 ,-0.42) | -0.31 (-0.32 ,-0.30) | <0.001 |
| TSHI | 1.12 (1.09 ,1.14) | 1.63 (1.60 ,1.65) | 1.94 (1.91 ,1.98) | 2.42 (2.39 ,2.44) | <0.001 |
| TT3RI | 4.84 (4.70 ,4.98) | 7.31 (7.06 ,7.56) | 9.37 (8.79 ,9.95) | 12.44 (12.04 ,12.84) | <0.001 |
| TT4RI | 8.66 (8.45 ,8.87) | 13.97 (13.62 ,14.33) | 18.93 (18.06 ,19.81) | 28.85 (28.05 ,29.64) | <0.001 |
| eGFR (mL/min/1.73m^2^) | 99.48 (98.07 ,100.88) | 95.97 (94.12 ,97.82) | 94.39 (92.50 ,96.27) | 90.13 (88.42 ,91.85) | <0.001 |

Continuous variables are described by weighted mean ± SD or weighted median (quartile spacing); description of adoption rate and constituent ratio of classified variables.

FT3: free triiodothyronine; FT4: free thyroxine; TSH: thyroid stimulating hormone; TPOAb: thyroid peroxidase antibody; TgAb: thyroglobulin antibody;

FT3/FT4: free triiodothyronine to free thyroxine ratio; TFQIFT4: Thyroid Feedback Quantile-based Index calculated by FT4; TFQIFT3: Thyroid Feedback Quantile-based Index calculated by FT3; TSHI: thyroid stimulating hormone index; TT4RI: total thyroxine (T4) resistance index; TT3RI: total triiodothyronine (T3) resistance index.
